# Porcine Epidemic Diarrhea Altered Colonic Microbiota Communities in Suckling Piglets

**DOI:** 10.3390/genes11010044

**Published:** 2019-12-30

**Authors:** Zhen Tan, Wanting Dong, Yaqun Ding, Xiangdong Ding, Qin Zhang, Li Jiang

**Affiliations:** 1 Key Laboratory of Animal Genetics, Breeding and Reproduction, Ministry of Agriculture, National Engineering Laboratory for Animal Breeding, College of Animal Science and Technology, China Agricultural University, Beijing 100193, China; tankoer@hainanu.edu.cn (Z.T.);; 2 College of Animal Science and Technology, Institute of Tropical Agriculture and Forestry, Hainan University, Haikou 570228, China; 3 Shandong Provincial Key Laboratory of Animal Biotechnology and Disease Control and Prevention, Shandong Agricultural University, Taian 271018, China

**Keywords:** colonic microbiota, porcine epidemic diarrhea virus, 16S rRNA gene, colonic mucosa, suckling pigs

## Abstract

Porcine epidemic diarrhea (PED) is a major gastrointestinal disease afflicting suckling pigs that causes huge industrial economic losses. In this study, we investigated microbiota from the colonic mucosa and content in healthy and PED piglets. High-throughput 16S rRNA gene sequencing was performed to identify inter-group differences. Firmicutes, Fusobacteria, Proteobacteria, and Bacteroidetes were the top four affected phyla. The proportion of Proteobacteria was higher in infected than in healthy piglets, and the opposite was observed for Bacteroidetes (more than four-fold higher in the healthy group). In the infected group, *Fusobacterium* accounted for 36.56% and 21.61% in the colonic mucosa and contents, respectively, while in the healthy group, they comprised 22.53% and 12.67%, respectively. The percentage of *Lactobacillus* in healthy colons (15.63%) was considerably higher than that in the disease group (<10%). In both the colonic mucosa and contents, functional enrichment differed significantly between healthy and diseased groups. Overall, infection with the PED virus increased the proportion of harmful bacteria and decreased the proportion of beneficial bacteria in the colons of piglets. Targeting intestinal microbiota could be a promising method for PED prevention, thus opening new avenues for future research.

## 1. Introduction

Porcine epidemic diarrhea virus (PEDV) is a member of the Coronaviridae family [1], and is known to cause enteric diseases in swine. Pathological changes include the infection of porcine enterocytes [2], acute mucosal damage, blunted intestinal villi, and reduced intestinal wall thickness [3]. Porcine epidemic diarrhea (PED) affects pigs of all ages, and causes clinical symptoms including severe enteritis, vomiting, acute diarrhea, anorexia, dehydration, and weight loss [4]. However, piglets are at the highest risk, with mortality rates as high as 80% for suckling pigs and nearly 100% for newborns [5]. In recent years, there were global increase in PED incidence, causing heavy losses to the pork industry [5,6,7,8]. For instance, a large-scale outbreak during October 2010 in Southern China led to over one million newborn piglet mortalities [9]. Studies on PED have mainly focused on the molecular characterization of PEDV [10,11], sequence-based phylogeny analysis, pathogenicity, the host immune response [12,13], and vaccinations. However, very little research has been performed on the relationship between PED and porcine gut microbiota. 

The gut microbiota plays important physiological, nutritional, and immunological roles in maintaining host health [14,15]. Microbes maintain the normal function of the intestinal mucosal barrier and contribute to immune system development [16,17,18]. Commensal microbes can prevent pathogenic invasions by competing for receptors and enteric nutrients [12], stimulating the innate immune system, producing antimicrobial compounds [16], and creating an anti-pathogenic microenvironment [12,19]. Therefore, increased knowledge on the gut microbiota community structure and functional capacity, especially in the large intestine, can contribute to a deeper understanding of host physiology and metabolism.

Previous studies have showed that PEDV induces gut microbiota dysbiosis in piglets and sows; this leads to decreased probiotic bacteria and increased pathogenic bacteria in the gut [1,20,21,22,23]. Most commensal bacteria, including Fusobacteria, Verrucomicrobia, and *Enterococcus*, were found to be more abundant in PEDV-infected pigs than in healthy ones [1,20,21]. Fresh fecal samples collected from healthy and PEDV-infected piglets revealed the structural segregation of microbial diversity disease status and age. Additionally, PEDV-induced dysbiosis increased the abundance of *Escherichia-Shigella*, *Enterococcus*, *Fusobacterium*, and *Veillonella*. The latter two genera also exhibited age-related increases among diarrheal piglets [23]. Some bacteria were common across all healthy piglets, but were not detected in diarrheal piglets. Moreover, healthy and PEDV-infected piglets differed significantly in clusters of orthologous groups (COGs) [23]. However, to the best of our knowledge, no research is available on the microbial structure and function of piglet colonic content and mucosa. Thus, the aim of this study was to thoroughly characterize these aspects in both healthy and PEDV-infected piglets. Our results are expected enhance the understanding of gut microbiota associated with PEDV. 

## 2. Materials and Methods

### 2.1. Animal Experiments

This trial was conducted at a commercial breeding pig farm in Shandong (Binzhou, China) using F1 offspring of Landrace and Yorkshire pigs. In January 2018, several piglets at the farm exhibited diarrhea and were tested to be positive for PEDV. Piglets with and without diarrhea symptoms were selected from sow stalls in the same farrowing house within 7–9 d after birth. Piglets were slaughtered to collect digesta and colonic mucosa tissues under aseptic conditions within 20 min. Samples were snap-frozen in liquid nitrogen until analysis. Piglets infected with PEDV were identified through quantitative real-time PCR (qPCR) using SYBR Green I and specific primers described previously [24], in a Roche LightCycler 480 instrument (Roche Diagnostics, Basel, Switzerland) following the manufacturer’s protocol. The results identified ten PEDV-infected pigs and eight healthy pigs. A total of thirty-six samples were collected from the colonic mucosa of healthy piglets (CoMH, n = 8), and infected piglets (CoMD, n = 10), the colonic content of healthy piglets (CoCH, n = 8) and infected piglets (CoCD, n = 10) (Supplementary Figure S1).

This work was performed in accordance with guidelines from the Quality Supervision, Inspection, and Quarantine of the People’s Republic of China (GB/T 17236–2008). All experimental procedures were approved by the Animal Welfare Committee of China Agricultural University (permit number: DK996) and were in accordance with the Guidelines for Experimental Animals of the Ministry of Science and Technology (Beijing, China). 

### 2.2. DNA Extraction

Microbial genomic DNA was extracted from the samples and purified using the QIAamp DNA Stool Mini Kit (Qiagen, Hilden, Germany) following the manufacturer’s protocol. Next, the DNA concentration was measured using a UV-Vis spectrophotometer (NanoDrop 2000; Thermo Fisher Scientific, Waltham, MA, USA), and the quality was confirmed with 1% agarose gel electrophoresis.

### 2.3. Amplification and Sequencing

The V4 region of the 16S rRNA gene was PCR-amplified using universal primers (515F-806R, forward, 5′-GTGCCAGCMGCCGCGGTAA-3′; reverse, 5′-GGACTACHVGGGTWTCTAAT-3′) combined with adapter and barcode sequences. 

The total reaction volume was 30 μL, containing 15 μL Phusion High-Fidelity PCR Master Mix (New England Biolabs, Ipswich, MA, USA), 0.2 μM forward and reverse primers, and 10 ng template DNA. The thermocycling protocol was as follows: 98 °C for 1 min; followed by 30 cycles of 98 °C for 10 s, 50 °C for 30 s, and 72 °C for 60 s; with a final extension at 72 °C for 5 min. The PCR products were purified using the GeneJET Gel Extraction Kit (Thermo Fisher Scientific) following the manufacturer’s protocol. Next, samples were pooled for high-throughput sequencing of bacterial rRNA genes using the Illumina Hiseq 2500 platform (2 × 250 paired ends).

### 2.4. Processing of Sequencing Data

To minimize the effects of random sequencing errors, raw fastq files were demultiplexed, quality-filtered using Trimmomatic version 0.33, and merged using FLASH version 1.2.7 [25,26]. To obtain high-quality tag sequences, initial base sites with Phred score <20 were truncated, tags were filtered out if their continuous high-quality base length was less than three-quarters of the whole sequence, and chimeric sequences were removed in UCHIME version 4.2 [27]. Sequences with ≥97% similarity were assigned to the same operational taxonomic units (OTUs) using USEARCH version 10.0 [28]. A representative sequence for each OTU was screened and taxonomically analyzed against the 16S rRNA database Silva, using Ribosomal Database Project Classifier version 2.2 [29]. Representative sequences were subjected to multiple sequence alignment and a phylogenetic tree was constructed. Next, the composition of each sample community was determined at the phylum, class, order, family, genus, and species level. 

### 2.5. Diversity Analysis and Functional Predictions

The Alpha diversity indices were evaluated using Mothur version 1.30 [30] and compared with Student’s *t*-test. The beta diversity per group was calculated using principal coordinate analysis (PCoA) based on weighted UniFrac distances [31,32] in QIIME version 1.8.0 [25]. Between-sample differences in evolutionary information were determined using the unweighted pair-group method with arithmetic mean (UPGMA) [33]. Images were drawn and statistical analyses were performed using R software. Linear discriminant analysis (LDA) Effect Size (LEfSe) was used to identify biomarkers that differed significantly between groups. Analysis of variance (ANOVA) was used to determine between-group differences in gut microbiota. Statistical significance level was set at *P* < 0.05.

Functional predictions for all OTUs were performed using the Kyoto Encyclopedia of Genes and Genomes (KEGG) and Clusters of Orthologous Groups (COG) databases, based on the PICRUSt-established structure of gastrointestinal microbiota [34]. 

## 3. Results

### 3.1. OTU Clustering and Taxonomic Composition

**Figure 1:**
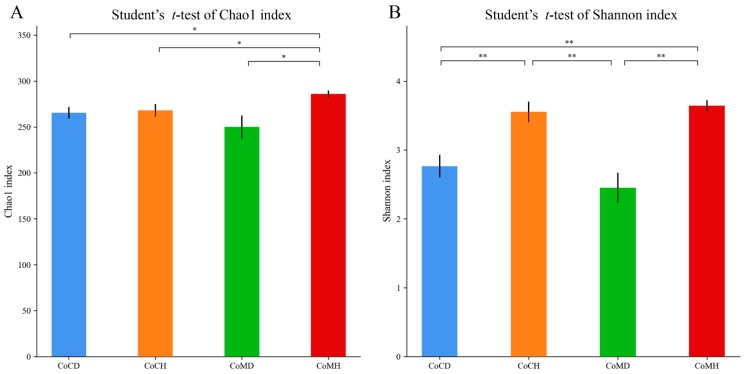
Dynamic changes in gut microbial alpha diversity (**A**, Chao1 index; **B**, Shannon index) of PEDV-infected piglets. Different letters denote significant differences in alpha diversity indices between groups based on the Student’s *t*-test and adjusted for false discovery rate (FDR) (* *P* < 0.05, ** *P* < 0.01).

We collected 1,616,525 quality-filtered and chimera-checked sequences with an average length of 440.25 bp. The mean number of reads per sample was 42,540, ranging from 31,732 to 56,822 reads. A good coverage of more than 99.9% was obtained for all samples, indicating reliable sequencing accuracy. We obtained a total of 316 OTUs, and 297 of these were observed in all four groups (Supplementary Figure S2). The Chao1 index was the highest in CoMH, while the Shannon index was higher in CoCH and CoMH than in CoCD and CoMD (*P* < 0.05) (Figure 1). 

**Figure 2:**
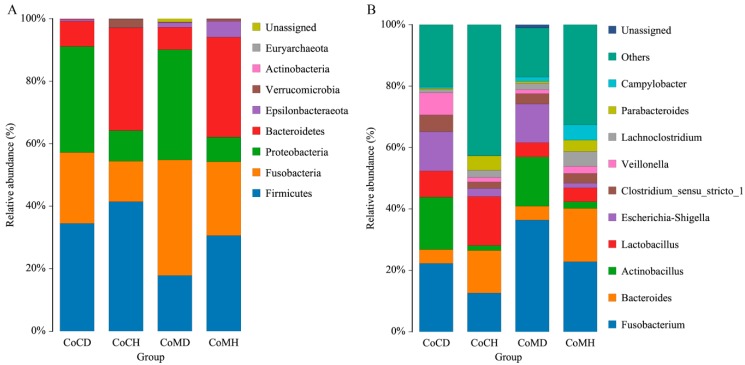
Histogram of the dominant phyla (**A**) and genera (**B**) in each group.

Taxonomic analysis indicated that Firmicutes, Fusobacteria, Proteobacteria, and Bacteroidetes were the top four phyla in all groups, representing approximately 95% of the sequences (Figure 2A). Microorganism structure differed more between healthy and infected groups than between colonic content and mucosa. The proportions of Firmicutes in colonic mucosa and content in the healthy piglets were 30.74% and 41.08%, respectively; while they were 17.86% and 35.00%, respectively, in the infected piglets. The proportions of Fusobacteria in colonic mucosa and content in the infected piglets were 37.15% and 22.05%, respectively; and 23.32% and 13.01%, respectively, in the healthy piglets. The proportions of Proteobacteria were higher in the infected colonic mucosa and content (35.02% and 34.15%, respectively) than that in the healthy groups (7.95% and 9.79%, respectively). In contrast to this general trend, the proportions of Bacteroidetes were more than four-fold greater in healthy groups (33.17% in CoCH and 32.04% in CoMH) than in the infected groups (7.99% in CoCD and 7.27% in CoMD).

Ten bacterial genera accounted for 70% of the total reads in all groups except CoCH (Figure 2B). Among healthy piglets, *Fusobacterium, Bacteroides,* and *Lactobacillus* were dominant, while in the infected piglets, *Fusobacterium*, *Actinobacillus*, and *Escheria–Shigella* showed higher relative abundances. The proportions of *Fusobacterium* in the infected groups were 36.56% (CoMD) and 21.61% (CoCD), whereas in the healthy groups they were 22.53% (CoMH) and 12.67% (CoCH). The *Bacteroides* percentages in the healthy piglets were 17.35% (CoMH) and 14.03% (CoCH), but only 4.54% (CoMD) and 4.49% (CoCD) in the infected piglets. The *Lactobacillus* proportion in CoCH (15.63) was considerably higher than that in the other groups. *Actinobacillus*, however, showed noticeably higher abundance in the infected piglets (17.21% in CoCD; 15.86% in CoMD) than in the healthy piglets (1.66% in CoCH; 2.26% in CoMH). The *Escherichia-Shigella* proportions were also higher in the infected groups (12.86% in CoCD; 12.61% in CoMD) than in the healthy groups (2.62% in CoCH; 1.58% in CoMH).

**Figure 3:**
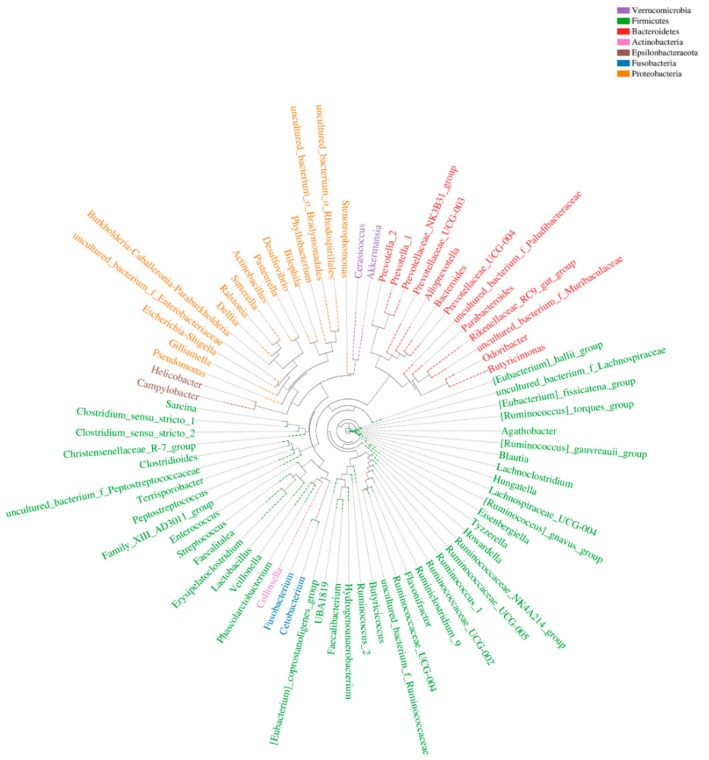
Phylogenetic tree of operational taxonomic units (OTUs) at the genus level. The ring shows the phylogenetic tree. The representative genus is in the same color as each phylum.

At each taxonomic level, we selected the most abundant OTU sequence as representative and performed multiple sequence alignment. We then constructed a phylogenetic tree from these data (Figure 3). Our analysis revealed that Firmicutes was the most well-represented phylum (by number of genera), followed by Proteobacteria and Bacteroidetes.

### 3.2. Variations in the Colonic Microbiota of the Healthy and PEDV-Infected Piglets

**Figure 4:**
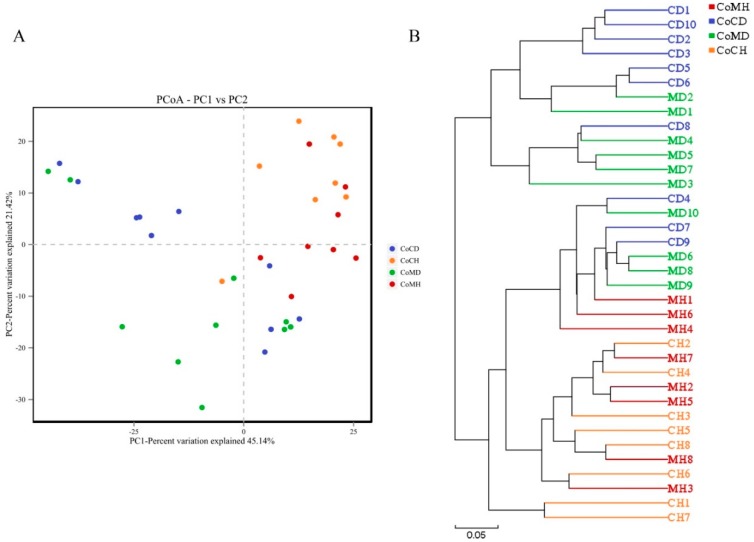
Principal coordinates analysis (PCoA) (**A**) and unweighted pair-group method with arithmetic mean (UPGMA) clusters (**B**). The phylogenetic tree resulting from these two methods demonstrate that healthy and infected colons contained different bacterial communities.

PCoA revealed variations between the microbiome profiles within each group based upon Bray Curtis dissimilarity (Figure 4A). Coordinate 1 (45.14% of variation) was associated with disease status (healthy or infected) and sample location (colonic content or mucosa). Samples from healthy and PEDV infected pigs showed more distant separation, while samples from the same group were more similar. Based on Bray Curtis distances, UPGMA showed that the microbes in healthy piglets were grouped in one cluster, while microbes in infected piglets were grouped in another in both colonic contents and mucosa samples (Figure 4B). Thus, the healthy samples were clearly distinguishable from the infected samples in the clustering tree, likely due to variations in their microbial compositions.

**Figure 5:**
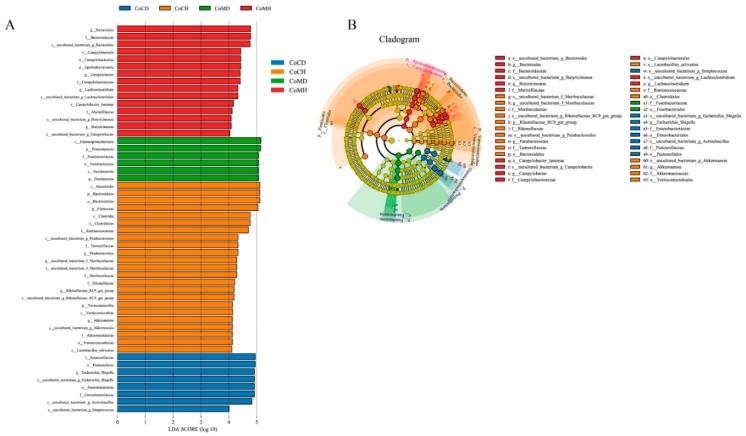
Colonic microbiota phylotypes differ between healthy and infected piglets. (**A**) Histogram of linear discriminant analysis (LDA) scores computed for differences in the proportions of colonic microbiota between healthy and diarrheal piglets. (**B**) Taxonomic cladogram of statistically and biologically consistent differences in colonic microbiota between healthy and diarrheal piglets. Taxa meeting an LDA significant threshold of >4 are shown.

Next, the LEfSe-identified biomarkers (Figure 5A) were found to be different between the four groups, as shown in the microbial cladogram (Figure 5B). We identified 52 different taxonomic groups (15 in CoMH, 6 in CoMD, 23 in CoCH, and 8 in CoCD) that could be used as biomarkers. More biomarkers were present in healthy groups than in infected groups. Microorganisms that differed between groups mainly included Firmicutes and Bacteroidetes in the CoCH and CoMH groups, Proteobacteria in the CoMD group, and class Gammaproteobacteria in the CoCD group. Eight phyla were shared between the four groups, and four phyla differed significantly between groups (*P* < 0.05, Wilcoxon rank-sum test; Supplementary Figure S3). The proportion of Proteobacteria was 3.92 fold higher in the infected groups than that in the healthy groups, while the proportion of Bacteroidetes was 4.25 fold higher in the healthy groups than in the infected groups.

### 3.3. Differences in Microbial Function between Healthy and PEDV-Infected Piglets

**Figure 6:**
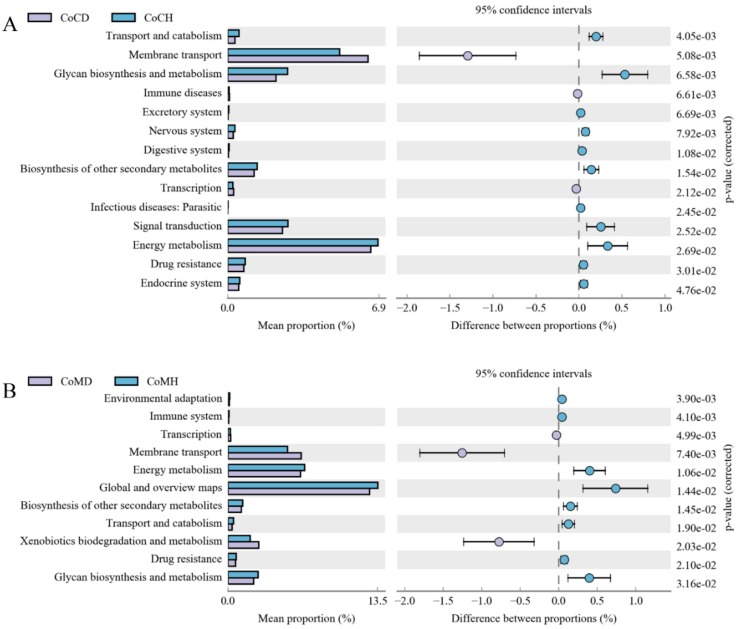
Comparison of enriched KEGG metabolic pathways in microbiota from colonic content (**A**) and from colonic mucosa (**B**) in heathy and PEDV infected piglets. The proportion of functional-abundance differences within the 95% confidence interval. KEGG, Kyoto Encyclopedia of Genes and Genomes.

A total of 43 second-level KEGG pathways were verified. Carbohydrate metabolism, Global and overview maps, and Amino acid metabolism were the top three functional annotations in all four groups, followed with Energy metabolism, and Metabolism of cofactors and vitamins. 14 pathways were identified for the colonic content (Figure 6A) and 11 were identified for the colonic mucosa (Figure 6B) between the healthy and PEDV-infected groups. Among them, 6 pathways were identified for both tissues. Additionally, Membrane transport and Transcription were more enriched in the disease groups than in the healthy groups.

**Figure 7:**
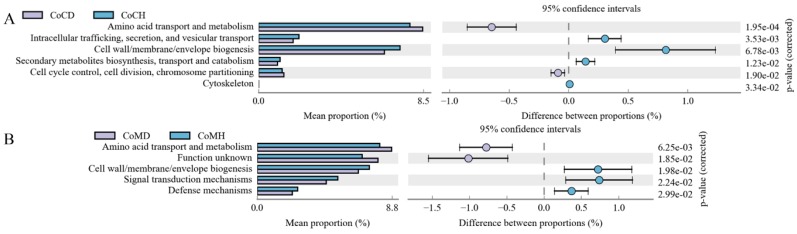
Comparison of COG functions in microbiota from colonic content (**A**) and colonic mucosa (**B**) in heathy and PEDV-infected piglets. Proportion of functional abundance differences within the 95% confidence interval. COG, Clusters of Orthologous Groups of proteins.

Through COG analysis, we obtained 24 second-level classifications across the four groups; the top annotations were General function prediction only, Carbohydrate transport and metabolism, Amino acid transport and metabolism, and Transcription. Six annotations differed between the CoCD and CoCH groups (Figure 7A), while five differed between the CoMD and CoMH groups (Figure 7B). In both colonic content and mucosa, Amino acid transport and metabolism and Cell wall/membrane/envelope biogenesis enrichment differed between the healthy and diseased groups.

## 4. Discussion

Several microorganisms, including Rotavirus, Coronavirus, Escherichia coli, Clostridium perfringens, C. difficile, Cryptosporidium spp., Giardia spp., Cystoisospora suis, and Strongyloides ransomi, have been linked to enteritis and diarrhea in suckling pigs. In this study, we successfully demonstrated that the microbial community structure of colonic mucosa and content differed significantly between healthy and PEDV-infected piglets. Likewise, previous research has shown that the proportions of Escherichia-Shigella, Enterococcus, Fusobacterium, and Veillonella increased significantly in PEDV-infected piglets, while those of short-chain fatty acid (SCFA)-producing bacteria (e.g., Rikenellaceae_RC9_gut_group, Butyricimonas, and Alistipes) underwent a decrease [21]. Moreover, the gut microbiota of PEDV-infected piglets was shown to exhibit significant changes at the genus and phylum levels [22] that are likely to disrupt microecological homeostasis. 

The intestinal microbial ecosystem of suckling piglets is unstable and sensitive to different external and internal factors in first week of birth. This sensitivity may generate individual differences in microorganisms, even in piglets from the same cohort [35]. Here, we found that PEDV infection significantly altered the gut microbiota in piglets, an outcome that was confirmed in several other studies [20,21,22,23].

Although the small intestine is the main site of PEDV infection, the large intestines are the main metabolic and absorption sites for microbial fermentation, and contain considerably greater microbial abundance than the small intestines. Therefore, studying the cecum and colon is necessary to obtain useful information for understanding the overall gut responses to PEDV infection. Microorganisms in the cecum and colon produce SCFAs that are beneficial to host health [36]. In our previous study, we compared the cecal microbial diversity between the healthy and PEDV-infected piglets and found that the diseases associated bacteria were increased along with a decrease of beneficial bacteria in the PEDV-infected piglets [37]. Similar results were observed for the colonic microbial diversity. According to the analysis of LEfSe, more bacteria were significantly enriched in cecal mucosa and cecal contents in the infected group than in the healthy group, while it was the opposite in the colon. In addition, differences existed in the distribution of some bacteria in the cecum and colon of the infected piglets. For example, the Fusobacteria was enriched in the colonic mucosa of the infected group in colonic position, while in cecum, this bacteria was higher in the cecal contents of the infected group than other three groups.

We observed a clear increase in the relative abundance of Proteobacteria and Fusobacteria in PEDV-infected suckling pigs. Fusobacteria increases have previously been observed in the feces of PEDV-infected suckling pigs [1,20,21]. Furthermore, many studies suggested that this phylum is associated with various clinical anaerobic infections (including in the mouth, teeth, gut, and brain) in humans, and is positively correlated with catarrhal appendicitis on the mucosal surface [38,39]. Within this phylum, the genus *Fusobacterium* plays a key role in promoting human colorectal cancer and various diseases in animals [40,41]. *Fusobacterium nucleatum* is important for the pathogenesis of various gut diseases, including intestinal inflammation and colon cancer [42]. A previous report showed that the abundance of *Fusobacterium* decreases sharply as the porcine gut microbiota matures from suckling to weaning [15]. The elevated *Fusobacterium* proportions in PEDV-infected piglets is likely correlated to colon inflammation.

The proportion of *Bacteroides* decreased in the colonic microbiota of PEDV-infected piglets. *Bacteroides* are important for carbohydrate fermentation and polysaccharide catabolism, as well as amino acid and protein utilization [43]. Several genera, including *Bacteroides*, *Prevotella*, and *Parabacteroides*, are important producers of SCFAs and are dominant in healthy piglet intestines [15,44]. SCFAs are rapidly absorbed by colonic epithelial cells [15], protect the host against colonic diseases, exhibit anti-inflammatory effects, and promote energy intake by intestinal fibers [45,46]. Thus, SCFA-producing bacteria contribute to carbohydrate fermentation, mucosal defense mechanisms, adipogenesis, and lipid oxidation [22,43]. Decreased *Bacteroides* abundance can result in reduced SCFA concentrations among PEDV-infected piglets, a link that could compromise intestinal and immune homeostasis. 

Here, LEfSe showed that each experimental group differed significantly in terms of microbial enrichment, suggesting that microbial profiles are useful as specific biomarkers for distinguishing between healthy and infected piglets (Figure 5). A number of the microbes identified as biomarkers are associated with intestinal diseases and piglet health. The relevant microbes mainly belonged to Firmicutes, Bacteroidetes, Proteobacteria, Fusobacteria, Verrucomicrobia, and Epsilonbacteraeota. Some bacteria from Firmicutes and Bacteroidetes were enriched in healthy piglets, while some from Proteobacteria and Fusobacteria were more abundant in PEDV-infected groups.

We observed similar outcomes when comparing cecal microbial diversity between healthy and PEDV-infected piglets. The abundance of butyrate-producing *Clostridium butyricum* was greater in the CoMH group than in the other groups. Another study found that *Butyricimonas* was present in all healthy piglets, but not in PEDV-infected piglets [23]. A reduction in this genus is linked to numerous autoimmune and inflammatory diseases, including inflammatory bowel disease, rheumatoid arthritis, and type 1 diabetes. Overall, these changes in biomarker bacteria compositions partly explained the low relative abundance of microflora related to energy metabolism, secondary metabolite synthesis, and amino acid metabolism in the intestines of PEDV-infected piglets. Collectively, our findings suggest that providing supplements to suckling piglets based on deficiencies of individual beneficial bacteria may prevent or alleviate PEDV-induced diarrhea.

Colonic microorganisms that differ in abundance between healthy and diseased groups are mainly involved in transport and energy metabolism. Therefore, PEDV likely causes negative effects by disrupting microecosystem homeostasis in porcine intestines. 

The timely treatment of PEDV-induced diarrhea and vomiting can effectively reduce mortality in newborn piglets [2]. Many previous researches [1,21,22,23] linking specific bacteria to healthy versus infected piglets suggests that regulating intestinal microbiota is a promising method for PED treatment. Indeed, gut microbiota have been successfully used to prevent diarrhea [47]. However, we noted that all infected samples in this study were PEDV-positive, so we could not determine the clinical importance of other detected viruses. To better characterize the intestinal microbiomes of healthy versus PEDV-infected piglets, we recommend that future studies fully examine virome diversity using a larger sample size and metagenomic de novo sequencing of the gut microbial genome.

## 5. Conclusions

Our study revealed the dysbiosis of colonic microbiota in PEDV-infected piglets. Infection increased the abundance of bacteria associated with diarrhea and other conditions, while decreased the abundance of beneficial SCFA-producing bacteria. Our results suggest that the gut microbiota is crucial for PED pathology and physiology. Overall, our findings greatly enhance the current understanding of PEDV-associated gut microbiota. 

## Data Availability

Data were deposited in the NCBI Short Read Archive (accession No. SRP162202).

## Supplementary Materials

The following are available online at https://www.mdpi.com/2073-4425/11/1/44/s1, Figure S1: Detection of PEDV in healthy groups and diarrhea groups using qPCR, Figure S2: Shared OTU analysis of the different piglet groups using Venn diagrams, Figure S3: Significant differences in microbe compositions between piglet groups, (* *P* < 0.05, ** *P* < 0.01).
