# Supplementary figures and images for: Porcine Epidemic Diarrhea Altered Colonic Microbiota Communities in Suckling Piglets
Source: Genes (Basel). 2019 Dec 30;11(1):44. doi: 10.3390/genes11010044 (PMC7016528; doi:10.3390/genes11010044)

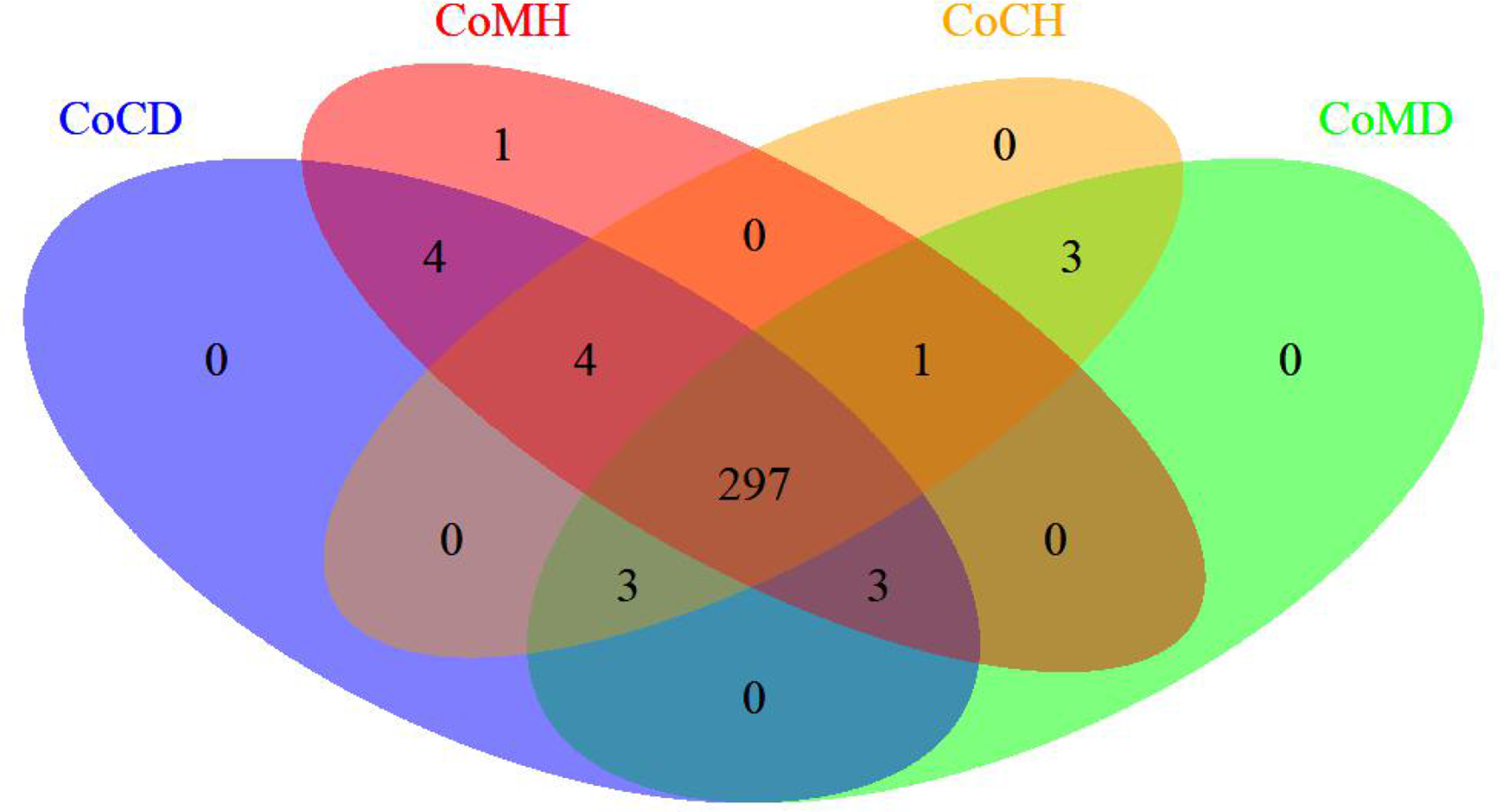

Supplement: Supplementary file 1 [file genes-11-00044-s001.zip › Figure S2.tif]

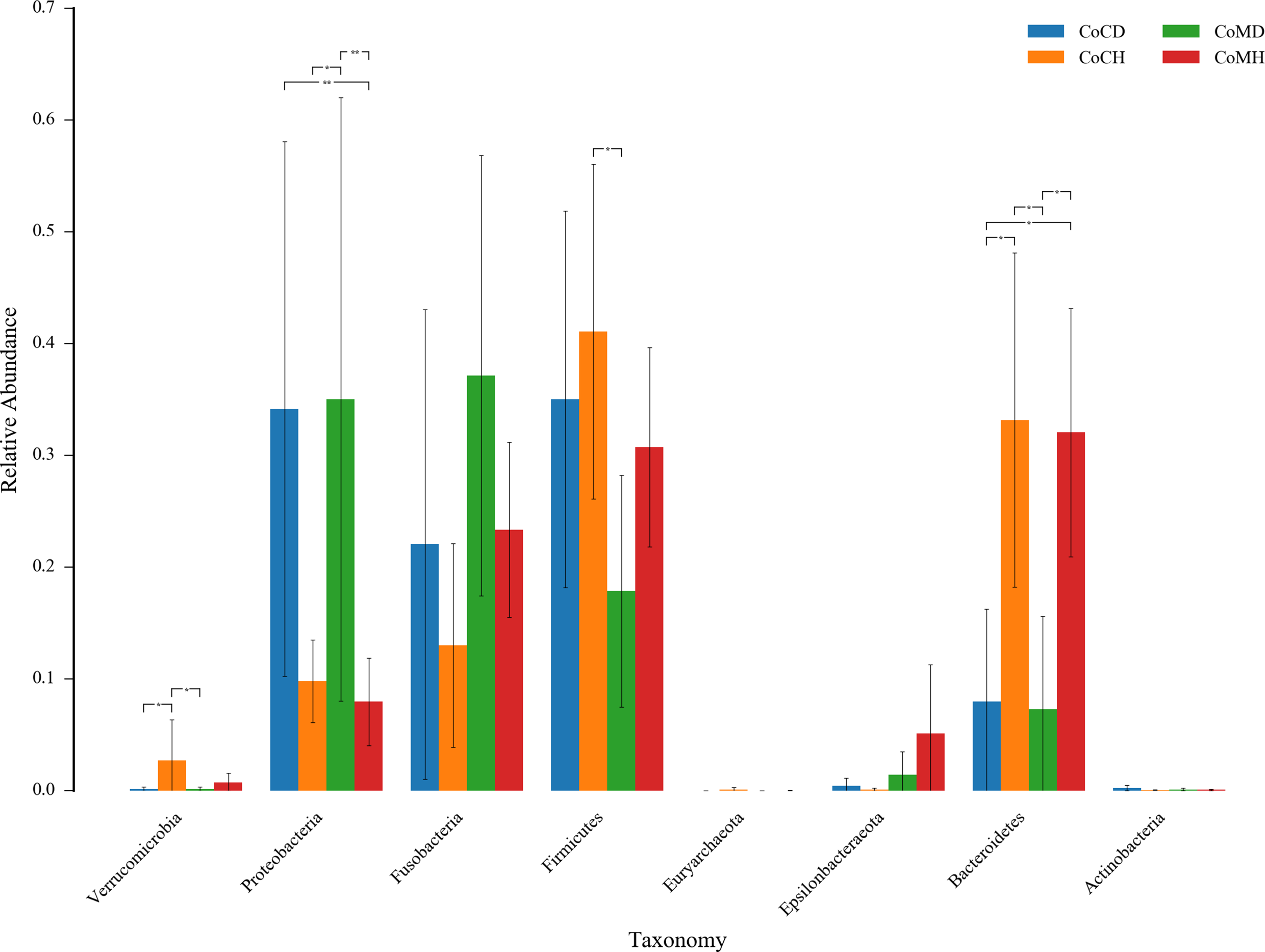

Supplement: Supplementary file 1 [file genes-11-00044-s001.zip › Figure S3.tif]

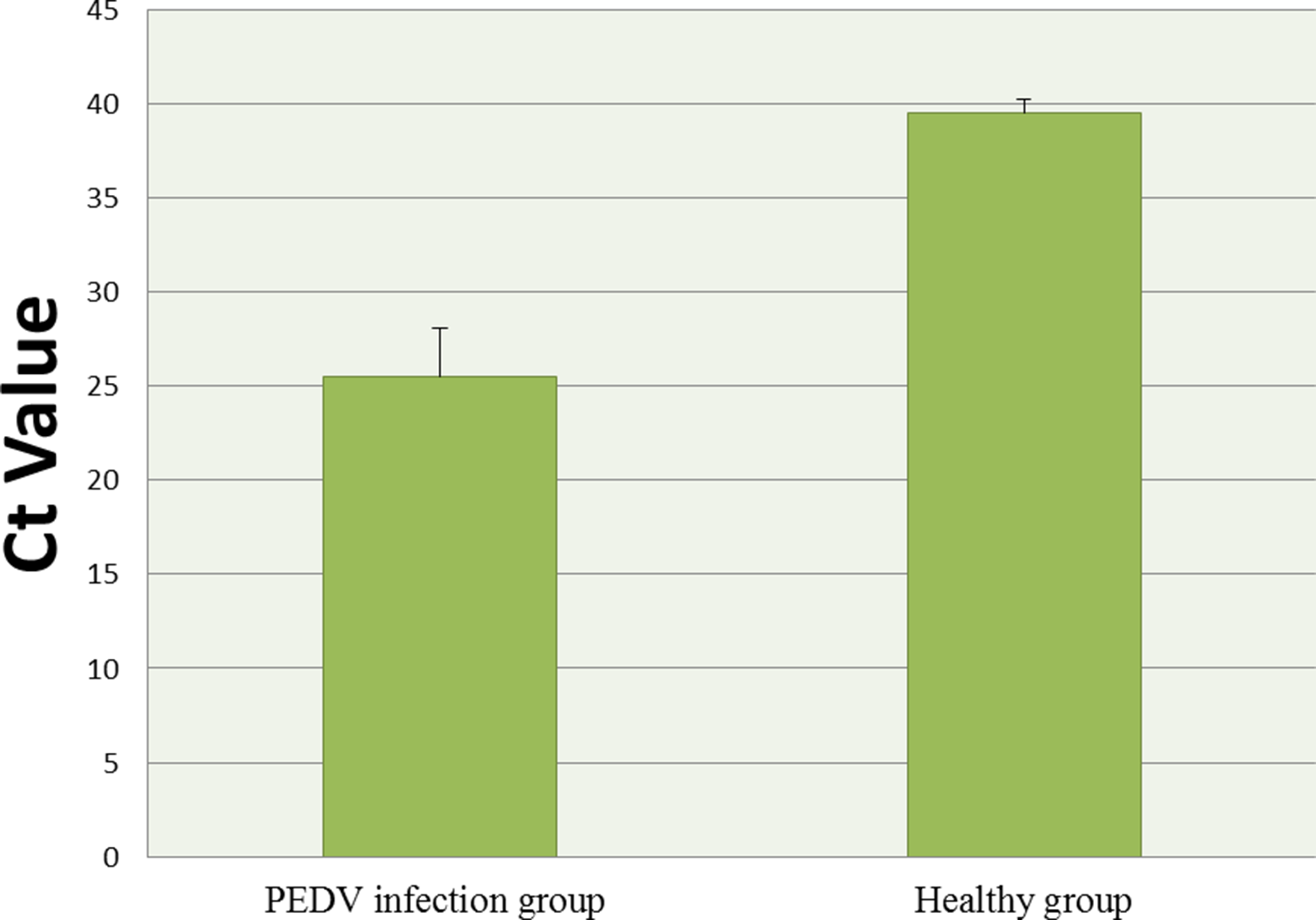

Supplement: Supplementary file 1 [file genes-11-00044-s001.zip › Figure S1.tif]
